# The Next Generation: How medical students use new Social Media to support their learning

**DOI:** 10.15694/mep.2019.000227.1

**Published:** 2019-12-10

**Authors:** Jonathan Guckian, Jessica Leighton, Richard Frearson, Laura Delgaty, Gabrielle Finn, Joanna Matthan

**Affiliations:** 1 Leeds Teaching Hospitals Trust; 2 Newcastle Upon Tyne Hospitals Trust; 3 Newcastle University; 4 Hull York Medical School

**Keywords:** Medical Education, Social Media, Technology Enhanced Learning, Snapchat, Instagram, Twitter, Blended Learning

## Abstract

This article was migrated. The article was marked as recommended.

Background

The rise of social media [SoMe] has changed medical education practice, possibly facilitating learning through conversational interaction, social feedback and relationships. Usage of newer SoMe tools like Instagram and Snapchat has not been scrutinised.This study aimed to understand how medical students may use newer SoMe tools, specifically Twitter, Instagram & Snapchat, in their learning, in the context of a parallel SoMe course.

Methods

An optional, parallel SoMe course was established at Newcastle University Medical School. 301 fourth-year medical students were invited to engage using Twitter, Snapchat and Instagram. Evaluation adopted a mixed methods approach, gathering SoMe analytics and survey data as well as qualitative, free-text responses from a questionnaire and focus-group discussion.

Results

Live-tweeting lectures featured 95 facilitator tweets, with five replies by students. 22 Instagram posts received no student responses, and three Snapchat stories were viewed 15,312 times, with 212 screenshots taken. Of questionnaire respondents, 75% [n=66] stated they engaged with content. Framework analysis of free-text responses and focus group discussion identified peer influence, fear of exposure, cognitive load and curiosity as drivers in new SoMe use.

Discussion

Medical students may engage with new SoMe for learning. This may manifest as yet another unilateral learning resource, rather than a tool for discussion or debate. Educators should be aware of external influences, such as peer influence, before assuming student interaction. Further research into medical student use of newer SoMe platforms is warranted, given their popularity, rapidly evolving nature and short lifespan.

## Introduction

The rise of social media (SoMe) has changed medical education practice, due to its ubiquitous nature and associated opportunities for innovation. 94% of medical students use some kind of SoMe platform ( Keenan, Slater and Matthan, 2018), with Facebook and Twitter established in the literature as commonly used by students. These platforms can support online communities of practice, as well as acting as an access point for recommended learning materials ( Cole *et al*., 2017). A prime example of the latter is the #FOAMed (Free Open Access Medical Education) movement, a global SoMe phenomenon ( Shah and Kotsenas, 2017).

Newer SoMe platforms and apps continue to emerge ( Table 1). Each vie for wider adoption within target audiences, adding complexity for the educational usage of these platforms due to the constantly evolving nature of SoMe. Instagram and Snapchat have made a vociferous entrance onto the SoMe scene: Snapchat reports over 300 million monthly users in 2019 ( Snapchat, 2019), and Instagram is only beaten in popularity by Facebook, with 1 billion monthly users ( Instagram, 2019). These platforms rely heavily on visual materials and, whilst there is evidence of medical education institutions ( Edinburgh Medical School, 2019), businesses and educators ( Inside the Boards, 2019) beginning to develop new SoMe medical revision resources, there has not been critical analysis of medical student use of these platforms. The importance of such scrutiny cannot be understated. Educators must anticipate whether Snapchat and Instagram will offer the same opportunities as Facebook or Twitter, or fall to the same fate as Vine, as transient trends in the fluctuating SoMe landscape ( Guckian and Spencer, 2018). Moreover, Facebook and Twitter have been criticised for generating professionalism concerns amongst staff and students and breeding ‘butterfly minds’ with shorter attention spans ( Delgaty, Fisher and Thompson, 2017): it remains to be seen whether such concerns impact upon new SoMe use.

**Table 1.** Glossary of Social Media Terms

Platform	Description
Twitter	Microblogging tool involving sharing of 280 character tweets, which may include polls, images or external links
Instagram	Visual based platform facilitating sharing of high-quality images. Allows discussion of images shared.
Snapchat	Mobile app in which users send photos, sometimes including drawings or cartoons, to individual friends or groups. Images deleted after at most 10 seconds, but can be shared to all friends for 24 hours, in the form of a ‘Snapchat Story’.
Facebook	Social networking tool allowing users to create profiles and share personal information with friends, family or colleagues

Developing an understanding on a diverse population’s SoMe behaviours is phenomenally complex. It is challenging to investigate behaviour in an environment where privacy is valued, and recently a legal requirement. It may prove difficult and ethically problematic to observe students in a ‘natural’ SoMe environment. However, there is evidence of older SoMe platforms being integrated with existing curricula, supporting small group learning, reflection and receipt of feedback from educators ( Sherbino and Frank, 2014). Observing newer SoMe platforms used in an artificial ‘parallel curriculum’ may offer insight into student Snapchat and Instagram use in supporting their learning.

**Figure 1.**
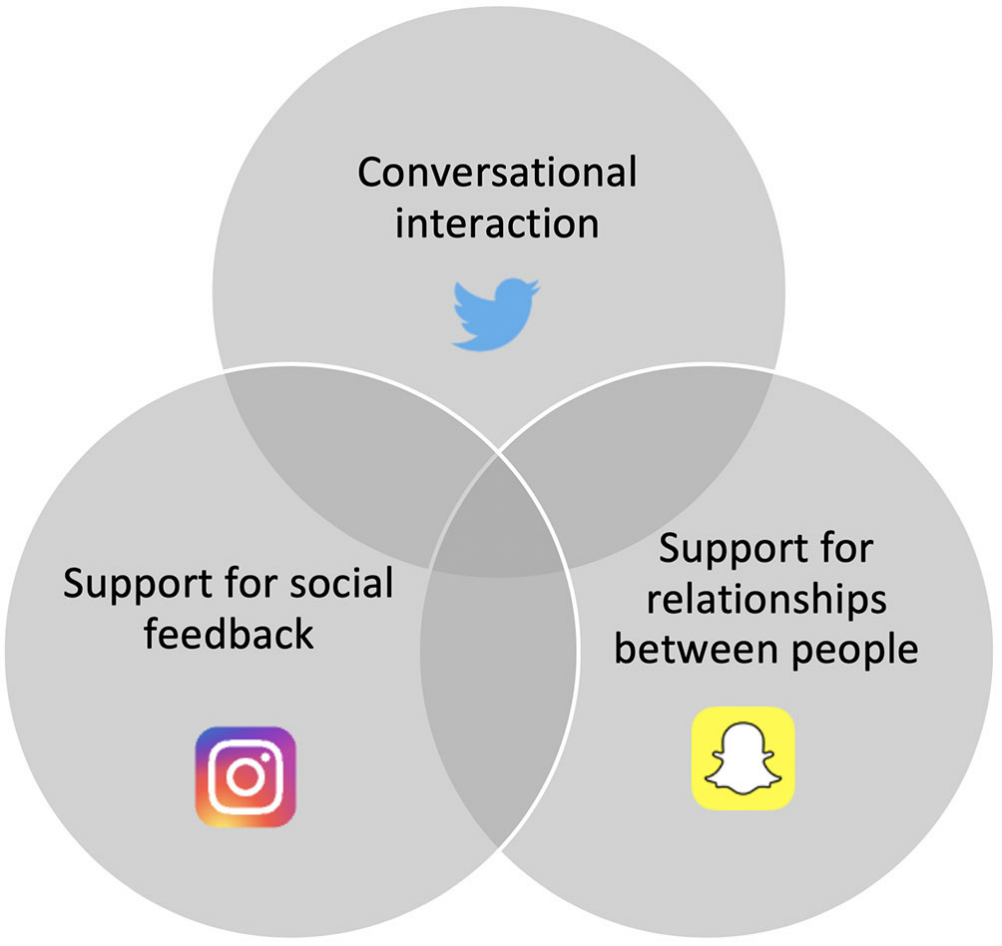
Framework for SoMe learning, adapted from Lee and McLoughlin (2010)

Whilst a comprehensive understanding of why students elect to use SoMe to support their learning is incomplete, the literature suggests that social constructivist theories may provide clues. One framework for SoMe learning proposed by Lee and McLoughlin (2010) relates to overlapping elements of conversational interaction, support for social feedback and support for relationships between people ( Figure 1) and creation of a supportive environment for meaningful interaction. To create a learning environment supportive of regular, positive SoMe interaction may require establishment of a digital community of practice ( Lave and Wenger, 1991). In the context of an integrated SoMe course, this may involve scaffolding by a facilitator and the use of appropriate SoMe tools to meet specific aims.

Adapting this principle to the SoMe landscape of 2019, individual platforms play specific roles in the framework. The text-based, microblogging nature of Twitter naturally facilitates conversational interaction. The primary role of text in Instagram is to respond to user-submitted creative media through comments. Snapchat facilitates one-on-one interaction through photos and videos, potentially supporting relationships between individuals.

The overarching aim of this study was to better understand if medical students are using newer SoMe tools (Twitter, Instagram & Snapchat) in their learning, and if so, how.

We aimed:

(1) to explore whether medical students would interact with new SoMe in a parallel course,

(2) to identify motivations and barriers to engagement with new SoMe,

(3) to establish why medical students use newer SoMe tools, specifically Instagram and Snapchat, to support their learning.

## Methods

### Parallel SoMe Course

A parallel SoMe course was established at Newcastle University Medical School for 301 fourth-year medical students. A course was chosen that was lecture-focused, well-attended and featured visual content suitable for a parallel SoMe programme. This course, Clinical Sciences and Investigative Medicine 2 (CSIM2), focuses on themed specialty weeks, and was partly organised and delivered by RF and JG. The SoMe course took place over one week, running parallel to the Multisystem Disease week in November 2017.

Three platforms were used: Twitter, Snapchat and Instagram, based on two years of pilot data collated by JG suggesting these were the most popular SoMe tools that this cohort of students would use to support their learning. Twitter, despite not being classed as ‘new’ SoMe, was included in this study due to its assumed value for conversational learning ( Lee and McLoughlin, 2010), in addition to reports in our pilot study that Twitter was a popular avenue for SoMe learning. The course facilitators were familiar with Twitter, feeling it would be best placed to advertise the technology and engage students, given its mutual popularity. It was felt that analysis of Twitter use would provide for a contextual understanding of the new SoMe use.

The hashtag ***#unofficialcsim2*** was used to uniquely tag SoMe content related to this course.

**Figure 2.**
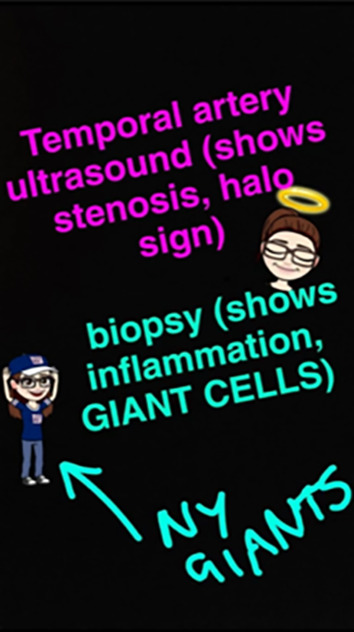
Example of a Snapchat Story post

‘Content’ was defined as one of three initiatives:

• Live-tweeting during CSIM2 lectures, sharing bite-sized learning points from lectures, links to relevant external resources and asking multiple choice questions through polls
• Sharing pre-prepared images on Instagram relevant to multisystem disease, and encouragement of students to create their own images for peer-learning
• Sharing Snapchat ‘stories’: collections of easily-made, ten second cartoon drawings highlighting learning points, posing questions and encouraging interaction ( Figure 2)

Posts were written in advance and in real time by JG and JL, who taught on the course and recently experienced the course as students. Input on posts was also received from course speakers. The SoMe account used was Medisense Medical Education ( Medisense Medical Education, 2019). This is a free online medical learning platform developed by JG, and was used for convenience, as many of the cohort were identified as being followers of the Medisense SoMe accounts.

### Study Design

This study utilised a mixed methods approach, gathering quantitative SoMe and survey data as well as qualitative, free-text responses from a questionnaire. Data were also collected utilising a focus group. This combined approach allowed formation of both an objective view of *#unofficialcsim2* use, as well as triangulation of data and a richer insight into student behaviour and motivations.

A focus group discussion with six students was carried out by two *#unofficialcsim2* facilitators. Both were unaffiliated to the university. One facilitator had previously taught on the course and one had been a student on CSIM2, albeit in a different cohort. Convenience sampling was used to recruit students, and recruitment was undertaken through email and SoMe. Methodologically, due to the lack of current understanding of the use of new SoMe, a post-positivist approach was used to first draw out basic, quantitative data, before gathering qualitative data to answer the most basic questions regarding this phenomenon.

### Data Collection and Analysis

Data were gathered from Twitter, Instagram and Snapchat, including discussions held between students or between students and facilitators. This data was gathered on each day of #unofficialcsim2, from data made available though ‘insights’ features of each SoMe platform. An online questionnaire was shared with students, both on SoMe and in person. As no validated questionnaires regarding SoMe use or behaviours were identified at the time of this study, a tailored questionnaire was created, using SurveyMonkey ( SurveyMonkey, 2019), asking questions regarding prior SoMe experience and engagement with #unofficialcsim2 content. Free-text responses were available for questions on motivations for interaction. Two pilot studies were undertaken using this questionnaire in 2015 and 2016 with similar cohorts to help test the survey.

A focus group was used to triangulate data so as to improve credibility. Discussions with the focus group centred on motivation for and barriers to engagement with #unofficialcsim2 and new SoMe for learning. The focus group discussion was recorded prior to analysis. Framework analysis ( Pope, 2000) was used to approach qualitative data, undertaken by JG and JL. Key themes were drawn from this data regarding motivation and barriers for #unofficialcsim2 engagement. Themes were devised from the data by JG and JL separately and then compared. Free-text responses and discussion points were indexed and interpreted to answer the study’s aims. The focus group discussions were transcribed and deidentified, and respondent validation of focus group transcripts was sought for credibility. Two independent educators, unconnected to the study, acted as observers of the audit trail for data analysis to improve dependability.

This study was approved by the Faculty of Medical Sciences Research Ethics Committee, part of Newcastle Universitys Research Ethics Committee.

## Results/Analysis

### Social Media Analytics

**Figure 3.**
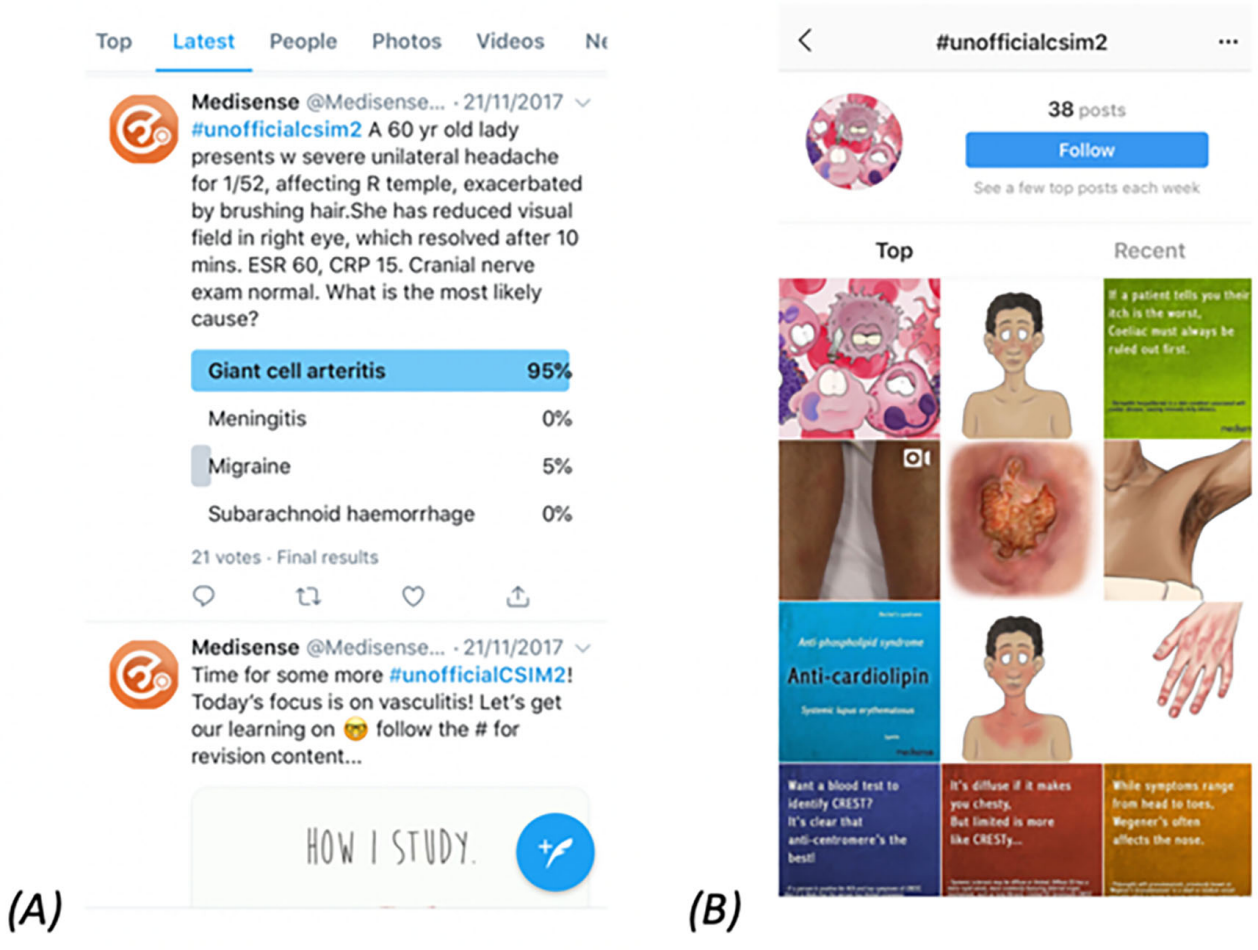
Timeline of Twitter (A) & Instagram (B) posts

These analytics cover all interaction with the SoMe accounts, with it being practically impossible to isolate data solely from our cohort of 301 students. The live-tweeting during lectures featured 95 facilitator tweets sent during four lectures. There were five tweet replies over the five day period. These replies were the only other accounts to tweet using the #unofficialcsim2 hashtag. There were 98 link clicks over the week, 82 likes and 42 retweets. Figure 3 demonstrates a search for #unofficialcsim2 content on Twitter & Instagram, demonstrating posts produced by the authors only and none by students.

There were 22 Instagram posts, with 0 replies and 38 likes. Data on link clicks was not available. There were 3 Snapchat stories, featuring 140 total images. These were viewed 15,312 times, with an average of 109 views per image and 212 screenshots. Three replies to questions posed by Snapchat stories were recorded. Snapchat content could only be viewed by direct followers of the Medisense account, of which there were 250, reducing the likelihood that these views came from external sources.

### Questionnaire Data

**Figure 4.**
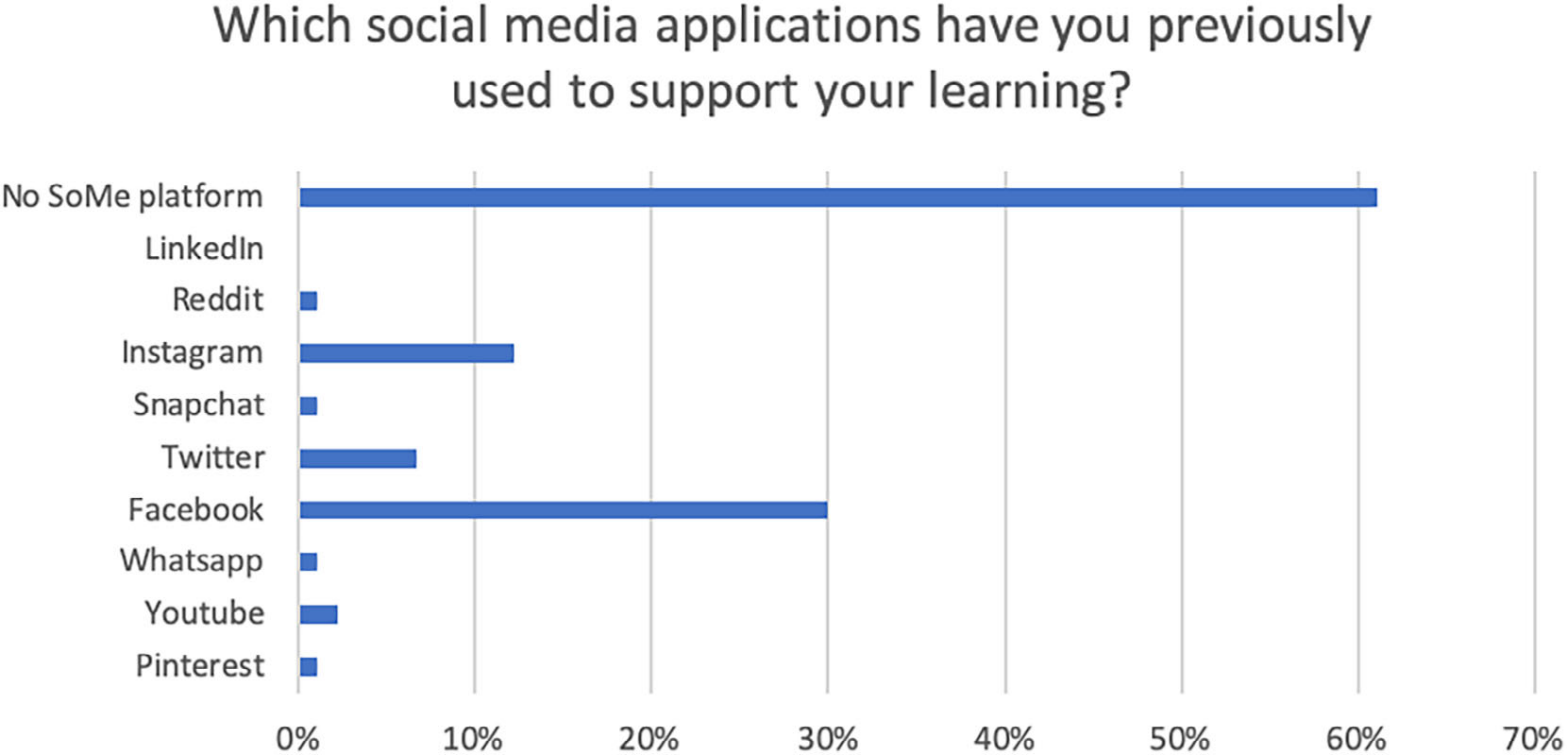
Student use of SoMe applications to support learning prior to #unofficialcsim2

94 responses were gathered from 301 students, a response rate of 31.9%. Concerning prior SoMe experience of the cohort for any use, 100% of students were Facebook users, 36% of students were Twitter users, 76% had used Instagram and 78% were Snapchat users. When considering SoMe platforms used for learning, most students stated they had never before used SoMe for learning ( Figure 4).

**Figure 5.**
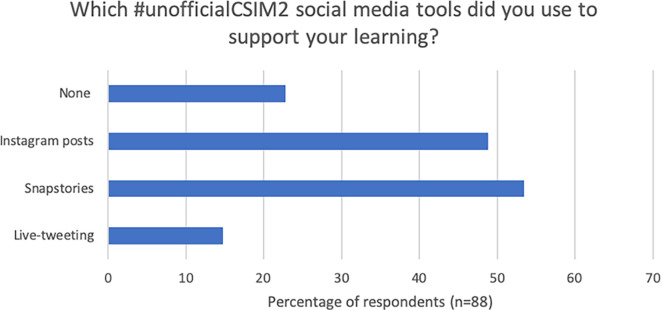
#unofficialcsim2 applications used to support learning in parallel curriculum

Of questionnaire respondents, 75% [n=66] stated they engaged with *#unofficialcsim2* ( Figure 5). Students were asked which of the resources they had used. ‘Used’ was defined as either read or replied to so as to support learning.

### Qualitative Data

Framework analysis of free-text questionnaire responses and focus group discussion was undertaken. Data were divided into two sections: ‘motivation for interaction’ and ‘barriers to interaction’. Five themes emerged: **peer influence**, **curiosity** and **convenience** were motivational themes, whilst **cognitive** **load**, **lack of induction** and **peer influence** (for a second time) emerged as barrier themes.

### Peer influence

Students described #unofficialcsim2 activity influenced by those in their peer group. Comments generally cited this as a positive influence, with statements such as ‘someone in my seminar group was looking at it during the seminar and I thought it looked helpful’ being commonplace. Comments described a sense of community, suggesting ‘live-tweeting is enjoyable and it.. brings everyone together’.

Students cited their peers as a major factor for reluctance to engage in discussions. Those who stated that they did not take part usually stated that their peers also had not engaged. The focus group revealed that SoMe can act as a ‘taboo topic.. our friends aren’t the kind of friends that discuss things on social media’.

### Fear

Some students described a fear of ‘missing out’. Platforms were described as ‘trendy’ and ‘popular’, whilst those who admitted to rarely posting content on Instagram stated that they had an account ‘because everyone does’. Students stated there was a difference between having an account and using it for learning.

Fear impacted upon open discussion. The public nature of SoMe discussion was suggested as a reason for lack of such discussion. Students expressed concerns of feeling ‘exposed’, identifying a risk that ‘there are people who will find’ previous posts and a perceived threat that this may ‘come back to bite you’. External warnings were expressed, including SoMe policies from medical schools and even warnings from medical school interviews. This could be contrasted with the private nature of Facebook Messenger, which students revealed was ‘the main way we communicated about seminars and exam revision’.

### Convenience

A motivating factor for students engaging with new SoMe was convenience. Students explained they could access content with minimum effort - ‘always on my phone so may as well’ - with a focus on unintentional learning: ‘seeing posts whilst scrolling through social media meant I was constantly revising.. even when it wasn’t my intention’. Brevity of content was viewed in a positive light: ‘the snapchats were short recap at the end’.

### Curiosity

The most commonplace motivation described for engagement with #unofficialcsim2 was that of novelty. Students frequently reported that they ‘wanted to try something new’, indicating they were not used to using such applications for their learning, or that the content seemed ‘enjoyable’ or ‘fun’. There were no comments indicating that students were curious about discussing content with each other or facilitators.

### Cognitive Load

A practical difficulty for engaging with the blended learning nature of #unofficialcsim2 appeared to be that it took place alongside ongoing lectures. Students described this as ‘overwhelming’, concerned that they may miss out on lecture content if they multi-tasked on SoMe. Students suggested this resulted in avoidance of live discussions during lectures and, instead, using tweets and snapchat as revision resources after lectures. Students felt they ‘had to choose’ between engaging with #unofficialCSIM2 and listening to lecture content. A ‘psychological barrier’ was described regarding viewing tweets after the event, as it was felt ‘the tweets were meant for live, “in the moment” learning’.

## Discussion

This study has demonstrated that, whilst students may engage with new SoMe to support their learning, the exact nature of that engagement is limited. Rather than involving themselves as part of a community with discussion and debate, students viewed the parallel SoMe curriculum as a unilateral facilitator-recipient relationship. ‘Engagement’ was seen to be viewing SoMe content in their own time, rather than during lectures or seminars, or following links to external resources. Students were heavily influenced by their peers when using new SoMe, whilst issues such as cognitive load and fear of exposure may have contributed. Cognitive load theory ( Young, Van Merrienboer, Durning, and Ten Cate, 2014) suggests a bottleneck for working memory, impacted upon by intrinsic load (essential aspects of the task), extraneous load (non-essential aspects) and germane load (deliberate use of cognitive learning strategies). The parallel course may have imposed a cognitive load that overwhelmed learners’ working memory. Students create SoMe identities, which may feature ‘virtual masks’ ( Finn, Garner and Sawdon, 2010); these are online veneers providing both anonymity and identity, distancing students from offline realities. Learners may alter their privacy settings and make distinctions between work and social practice on SoMe. Such factors may impact upon the peer influence and fear of exposure experienced by our students, driving the unilateral relationship with SoMe.

Whilst this investigation centres on understanding new SoMe, it has educational implications for all similar platforms. SoMe has been advocated as providing opportunities for enhancing educational value due to its potential for formation of relationships and conversational interaction ( Lee and McLoughlin, 2010). It has been claimed SoMe encourages shy students to engage more, allows staff to answer questions during lectures and hands control to students ( Forgie, Duff and Ross, 2012). Several studies (Davis *et al*., 2015, Cheston, Flickinger, and Chisolm, 2013) make broad claims that the use of SoMe for learning is ubiquitous and seemingly ensures student-centred learning, due to ‘going where the students are’.

This study directly contradicts the aforementioned observations, suggesting that due to numerous complex external pressures, modern students may not feel comfortable with SoMe debate, particularly in a public forum utilising new SoMe. Our findings, instead, suggest that learners may see SoMe as a tool for unilateral learning, much like a textbook or a podcast. For educators considering including SoMe in their teaching, we suggest a more thoughtful approach may be warranted, particularly as distinctions between different, newer SoMe platforms are rarely established in the literature. SoMe trends evolve rapidly, with platforms dying off as quickly as they rise ( Guckian and Spencer, 2018), meaning assumptions of regular usage may not be justified.

Research suggests that the average person has seven SoMe accounts ( Mander, 2016). Our findings resonate with evidence that a ‘Fear of Missing Out’ (FOMO) leads to increased SoMe usage ( Blackwell *et al*., 2017); however, this does not guarantee what that usage looks like in practice. This study recommends that educators pause before engaging their own FOMO and challenge assumptions that simply posting on new SoMe will lead to learning through discussion.

Whilst studies have identified that medical students and educators use multiple SoMe tools ( El Bialy and Jalali, 2015, Patel, Yazd and Dellavalle, 2017) this is the first to critique the use of Snapchat and Instagram as medical education resources. It is the first to investigate student engagement with multiple SoMe platforms as adjuncts to a traditional medical school curriculum. It argues against a widely-held assumption that the use of SoMe in medical education guarantees learning through discussion, implying that the reality of learning on new SoMe is a complex web of external influences which are challenging for the educator to manoeuvre.

### Limitations

Whilst this study has value in demonstrating the behaviours of students when engaging with new SoMe, there are limitations. Students may use SoMe differently in an environment not specifically tailored to promote learning through these platforms. Further research is required to better understand the difference between public and private use of SoMe for learning.

The most favoured SoMe tool of this cohort, Facebook, was not included as a platform for learning and dissemination of resources. This was based on two years of pilot data suggesting that these students did not use Facebook to support their learning, further underlining the idea that SoMe trends fluctuate rapidly ( Villanti *et al*., 2017). A limitation is that analytics data may include views, likes or retweets from individuals not in the study cohort, due to the practical challenges of isolating content to this cohort alone.

Understandable concern has been raised regarding conflation of reading of SoMe content with deeper learning that comes from debate with colleagues or in-depth study of research ( Weiner, 2015). Further research is required to understand the long-term impact of live-tweeting or similar initiatives on academic performance.

Finally, the environmental context of #unofficialcsim2 must be taken into consideration as a limitation. Salmon’s model of online learning ( Salmon, 2012) emphasises the importance of detailed induction to technology for learners: without such introduction, it is challenging for students to have the skills or motivation to advance to online socialisation or knowledge construction. Whilst the parallel SoMe course was highlighted numerous times to students over the week, no formal induction workshops were undertaken and students may have found as a deterrent to engagement. Moreover, whilst the multi-system disease course was chosen as it was felt to be visually appealing and challenging, it took place a fortnight before an important assessment period for these students, potentially adding to the cognitive load.

## Conclusion

This study has identified that medical students may engage with new SoMe for their learning. However, this engagement may manifest as yet another unilateral learning resource, rather than a tool for discussion or debate. Educators should be aware of external influences such as peer influence, cognitive load and fear of exposure before assuming student interaction on SoMe, whilst further research into medical student use of newer SoMe platforms is warranted, given their widespread use.

## Take Home Messages

• New Social Media platforms are already being used by students and educators to support learning.
• Using new Social Media as part of a learning programme does not guarantee an effective forum for discussion or debate.
• Students may be driven off Social Media interaction for learning by peer influence, cognitive load and fear of exposure.

## Notes On Contributors

Dr Jonathan Guckian (ORCID ID: https://orcid.org/0000-0002-8162-1583) is a Core Medical Trainee in Leeds. He is also Director for Social Media & Communications at the Association for the Study of Medical Education (ASME) and founder of Medisense Medical Education, an online platform for creative medical education learning resources. His interests include Social Media in medical education and flexible postgraduate training.

Dr Jessica Leighton is a Foundation Doctor at Newcastle Upon Tyne Hospitals Trust, with academic interests in hepatology and medical education. She is the undergraduate lead for Medisense Medical Education, coordinating the production of medical education resources.

Dr Richard Frearson is a consultant geriatrician/physician and Clinical Sub Dean for Tyne Base Unit and Course Director for Clinical Sciences and Investigative Medicine at Newcastle University. He has a broad interest in all issues required for the management of undergraduate education across health and education sectors with a special interest in the skills required for the teaching of clinical reasoning to students.

Dr Laura Delgaty is Deputy Degree Programme Director and a personal reader in medical education at Newcastle University School of Medical Education, with an interest in both curricular studies and technology.

Professor Gabrielle Finn (ORCID ID: https://orcid.org/0000-0002-0419-694X) is Chair in Medical Education and Director of the Health Professions Education Unit. Gabrielle is a National Teaching Fellow with an interest in gender, professionalism and pedagogy.

Dr Joanna Matthan is Senior Lecturer and Director of Academic Studies at the School of Dental Sciences, Newcastle University, UK. She has a background in Medicine and English and a previous career in the corporate world. She predominantly teaches anatomy to dental and medical students but also has an interest in technology-enhanced learning, clinical reasoning, interprofessional education and introducing teaching improvements that facilitate deeper learning to students. Her research interests range from anatomical themes to wider clinical education ones ranging from simulation to digital technology usage.

## Declarations

The author has declared the conflicts of interest below.

Jonathan Guckian is Founder and a Director of Medisense Medical Education Ltd, the social media platform used to share content in this study. Neither Medisense nor JG supplied funding for this project and there was no financial gain from this study for any party, either directly or indirectly. Jessica Leighton is a volunteer for Medisense.

## Ethics Statement

This study was approved by the Faculty of Medical Sciences Research Ethics Committee, part of Newcastle University’s Research Ethics Committee. This committee contains members who are internal to the Faculty, as well as one external member. This study was reviewed by members of the committee, who must provide impartial advice and avoid significant conflicts of interests.(No Ethics Approval Reference Number supplied at Institution)

## External Funding

This article has not had any External Funding
