# Supplementary material for: The Next Generation: How medical students use new Social Media to support their learning
Source: MedEdPublish (2016). 2019 Dec 10;8:227. [Version 1] doi: 10.15694/mep.2019.000227.1 (PMC10712506; doi:10.15694/mep.2019.000227.1)
Supplement: Peer review history

# Reviewer report: Reviewer response for version 1

**DOI:** 10.21956/mep.19943.r30278

**Published:** 2019-12-29

**Authors:** Catherine Hennessy

**Affiliations:** 1 Brighton Sussex Medical School

This review has been migrated. The reviewer awarded 4 stars out of 5 This is a timely article giving rare insight into the motives and barriers for why medical students, particularly end-stage medical students, engage with tailor-made social media educational tools. I was particularly interested in the qualitative responses concerning the barriers for engaging in social media educational tools, such as fear of exposure and cognitive load, as I think these have been suspected by educators previously but rarely voiced by students themselves. This article certainly creates an argument for educators not to jump on the social media bandwagon with the belief that this is what students want. Perhaps students are capable of deciding how, when and why they wish to use social media for their own personalised learning rather than it being pushed on them.I did have some concerns as follows:The social media tools were only used for 1 week – is this really enough time for students to settle in to using these tools given that students are often working a week or two behind what is running in the curriculum. It may have been that the students would have valued the social media tools continuously throughout a term so they had more of a chance to figure out how it could work for them and their learning pattern/revision timetable.Also, there was very little information on how the social media tools were “advertised” to the cohort and whether they were given any instruction or ideas on how it could be used/ how they could engage or contribute to it. This may be the reason why there was a lack of replies etc and would also explain the lack of induction barrier. This barrier was not discussed and I would have liked to hear more about it. It might raise the issue that students are not sure how to start using social media for educational reasons and may need some guidance. Perhaps educators assume that students are confident to start using social media for their education but they may require some more guidance than we realise.

Reviewer Expertise:

NA

No decision status is available

# Reviewer report: Reviewer response for version 1

**DOI:** 10.21956/mep.19943.r30275

**Published:** 2019-12-17

**Authors:** Simon Fleming

**Affiliations:** 1 Barts and The London

This review has been migrated. The reviewer awarded 4 stars out of 5 A fascinating paper, unpacking the new and innovative ways learners are accessing knowledge. It's great to see a piece of research involving multiple stakeholders in education as authors. From my perspective, the "conflict" around the use of MediSense was declaredPlus, if one reviews the take home messages, i'm pretty sure these are relevant and valid outcomes. - New Social Media platforms are already being used by students and educators to support learning.- Using new Social Media as part of a learning programme does not guarantee an effective forum for discussion or debate.- Students may be driven off Social Media interaction for learning by peer influence, cognitive load and fear of exposure.I would love to see if the authors have any rebuttal to some of the comments on social media and here - some of which I am inclined to agree with (around language and clarity of data) and some of which I am inclined not to (the role of industry/platforms/force in the study)

Reviewer Expertise:

NA

No decision status is available

# Reviewer report: Reviewer response for version 1

**DOI:** 10.21956/mep.19943.r30279

**Published:** 2019-12-17

**Authors:** Ken Masters

**Affiliations:** 1 Sultan Qaboos University

This review has been migrated. The reviewer awarded 1 stars out of 5 An interesting paper on how medical students use new social media to support their learning.I do, however, have problems with the role of Medisense in this research, and, unfortunately, most of this review focuses on this issue. When a private company’s products are used in education institutions’ research, the situation is always delicate, and their roles must be clear and crucial to the research:• I’m uncomfortable about the overall relationship between Medisense, the course and the institution, and the value of using Medisense. Did students need to follow the Medisense Twitter and Instagram accounts to access the material?o If yes, this is disconcerting, as Medisense Ltd is a private company, not affiliated to the institution, and there is no benefit (for the students) for requiring the students to follow these accounts (apart from the benefit to Medisense by increasing the number of followers). o If no (i.e. the hashtag was enough), then why were Medisense accounts used for the posting; there was no need – the researchers should rather have used either their own accounts or created a course account.• This is especially important as the lead author’s institution in the paper is “Leeds Teaching Hospitals Trust” and we discover only in the notes on authors and the conflict of interest that the lead author is the founder and a Director of Medisense. Although there was no financial gain for Medisense, it would have benefitted from the exposure to the students (as is evidenced by the tweets in Figure 3), and there appears to be little reason for this. The company also benefited from using the company accounts because all their other followers would have witnessed the research. (The lead author has since tweeted about this article through his private Twitter account, and it has been re-tweeted through the company account, so acting as an advertisement).• I am not accusing the authors of abusing the situation for Medisense’s gain, but the paper has not adequately explained the relationships and the need for using the Medisense accounts and platform. The paper ostensibly evaluates students’ use of three SoMe platforms Twitter, Snapchat and Instagram, focuses on these, and then, unexpectedly, introduces Medisense, and their accounts are used for no apparent reason. Given the three SoME platforms under study were Twitter, Snapchat and Instagram, data were gathered only from these, and the survey was run through SurveyMonkey, why was the Medisense platform used at all?• The paper says that the Medisense platform was used “as many of the cohort were identified as being followers of the Medisense SoMe accounts.” I would like to see numbers on this, as “many” is too vague (The company may have benefited from a boost in their SoMe followers) and this still does not explain why the platform was used if the hashtag was used, and the all the results came from the SoMe platforms.• I see that the students were forced to follow the Medisense Snapchat account to access the Snapchat material (250 of the 301 students “chose” to follow the company account). This, again, has problems of convolution, and the authors should have used either one of their professional accounts, or created a special course account.Other issues:• “a legal requirement” The authors are possibly referring to GDPR, but this needs to be made explicit.• Many Results are given as percentages only, and should be given both as raw numbers and percentages, to at least one decimal place.So, while the paper is interesting, the role of Medisense is unclear, and appears to have served little purpose for the research. Mentioning it in the protentional conflict of interests is good, but does not change the fact that Medisense derived benefit from the process, and the authors need to describe its meaningful value to the research, which, from the aims and descriptions, could have been carried out without using its SoMe accounts or platform. This is reinforced by the fact that the Discussion does not refer to its role, as it does not appear to have been of material value to the research or the insights – it appears to merely have been the conduits for the SoMe accounts that should rather have been the teachers’ or specially-created course accounts. I look forward to Version 2 of this paper in which these issues are clarified.

Reviewer Expertise:

NA

No decision status is available

# Reviewer report: Reviewer response for version 1

**DOI:** 10.21956/mep.19943.r30277

**Published:** 2019-12-13

**Authors:** P Ravi Shankar

**Affiliations:** 1 American International Medical University

This review has been migrated. The reviewer awarded 4 stars out of 5 This is an interesting article where the authors explore the use of newer social media platforms by medical students. They highlight the advantages of some of these platforms. Privacy concerns of students and the fear of violating the social media use policy of the institution have also been mentioned. The challenges of live tweeting have also been mentioned. Peer group seems to be an important influence on the use of social media platforms. Widespread access to the internet and these platforms can enable these to be used in resource limited settings also.

Reviewer Expertise:

NA

No decision status is available

# Reviewer report: Reviewer response for version 1

**DOI:** 10.21956/mep.19943.r30276

**Published:** 2019-12-11

**Authors:** Felix Silwimba

**Affiliations:** 1 University of Lusaka

This review has been migrated. The reviewer awarded 5 stars out of 5 this is an excellent article to capture the needs of modern learners. modern learners rely more on use of IT. it is worth trying out in low-middle income countries

Reviewer Expertise:

NA

No decision status is available
